# Changes in Circulating Levels of Long Non-Coding RNA p5549 and p19461 Following Metabolic Bariatric Surgery (MBS): A Prospective Study

**DOI:** 10.1007/s11695-024-07596-5

**Published:** 2024-12-09

**Authors:** Shaimaa Ammar, Tamer Abdelbaki, Bassma Elsabaa, Hoda El Assi, Heba Kassem

**Affiliations:** Alexandria University, Alexandria, Egypt

**Keywords:** Obesity, Bariatric surgery, Metabolic surgery, Epigenetics, LncRNA, Realtime PCR

## Abstract

**Background:**

Obesity is attributed to a combination of factors such as lifestyle, environmental influences, and genetic background. Nowadays, the issue of obesity has grown to an epidemic scale. Environmental changes, having contributed to the sharp rise in obesity prevalence, are not the only contributing etiologic factors. Inherent biological variables interact with environmental factors resulting in obesity. Epigenetic mechanisms may explain part of obesity heritability. One of the recently discovered epigenetic mechanisms for controlling gene expression is long non-coding RNAs (lncRNAs). Circulating lncRNA p5549 and p19461 levels were reported to be significantly lower in individuals with obesity. This study aimed to evaluate whether weight loss following metabolic/bariatric surgery (MBS) can be related to altered expression levels of those lncRNAs, which have been reported to be reduced in individuals with obesity.

**Methods:**

Comparison of circulating levels of lncRNA p5549 and p19461 before and 12 weeks after MBS in thirty-four patients was conducted to evaluate whether MBS can revert the altered levels of these lncRNAs. None of the participating patients were lost to follow-up, and all underwent re-evaluation of post-surgical expression levels.

**Results:**

lncRNA p5549 expression levels in serum were found to increase significantly in the postoperative samples compared to preoperative samples (fold increase: 4.63 ± 7.68, p = 0.014).

**Conclusion:**

Epigenetic changes in patients with obesity, specifically lncRNA-p5549 expression levels, are reversed after MBS. The postoperative increase in the expression levels of lncRNA- p19461 was not statistically significant.

## Introduction

The rising global prevalence of obesity is a significant health concern, with the World Health Organization (WHO) reporting that over 1 billion people worldwide suffer from obesity[1]. According to the "Guidelines for the Management of Overweight and Obesity in Adults," weight loss treatment is recommended for 64.5% of adults diagnosed with obesity[2]. Various weight loss strategies are available, starting with lifestyle changes aimed at achieving a negative energy balance through diet, exercise, and behavior modification[3]. Pharmacotherapy is advised for individuals with a BMI ≥ 30 kg/m^2^ or a BMI ≥ 27 kg/m^2^ with obesity-related comorbidities, when lifestyle changes alone are insufficient [4–7]. Few Obesity Management Medications (OMM) are currently available for polygenic obesity, including centrally acting, peripherally acting, as well as dually- acting drugs like Glucagon- Like peptide- 1 (GLP-1) receptor agonists as Semaglutide, which can help patients lose more than 15% of their body weight when combined with behavioral therapy. Phentermine-Topiramate extended-release, Liraglutide, Bupropion/Naltrexone sustained release, and Orlistat have also been approved for long- term management of obesity.[4, 8]. Tirzepatide, an anti- diabetic medication, was recently approved for obesity management. It is a GLP-1 receptor agonist and a glucose-dependent insulinotropic polypeptide (GIP) that decreases appetite and subsequently food intake. Tirzepatide provided clinically meaningful weight reduction, and its safety was similar to other GLP-1 receptor agonists [9].

The response to OMM is frustratingly heterogeneous. This heterogeneity may be attributed to pharmacogenomics, which refers to differences in drug metabolism, absorption, and effect in various patients because of differences in their genetic makeup e.g., in patients treated with Topiramate, variants in the insulin receptor gene (INSR) have been associated with differences in weight loss among them [10]. Also, variants in Glucagon-like peptide-1 receptor gene (GLP-1R) have been linked to differential response to Liraglutide [11].

The currently- licensed OMM are less effective in reducing weight than Metabolic and bariatric surgery (MBS). Additionally, the challenging delivery of centrally acting drugs, the restricted efficacy of the peripherally acting Orlistat, as well as the absence of long- run safety data on the dually- acting Liraglutide, Semaglutide and Tirzepatide are points to be considered [8].

MBS is recommended for individuals with a BMI greater than 35 kg/m^2^, regardless of the presence, absence, or severity of comorbidities, and for patients with type 2 diabetes (T2D) who have a BMI greater than 30 kg/m^2^. Additionally, MBS should be considered for individuals with a BMI between 30 and 34.9 kg/m^2^ who do not achieve substantial or long-term weight loss, or improvement in comorbidities, through non-surgical methods [12]. The most common procedures are laparoscopic sleeve gastrectomy (LSG) and laparoscopic Roux-en-Y gastric bypass (LRYGB)[13]. These surgeries are effective and sustainable treatments for severe obesity, often improving or inducing remission of related medical issues, possibly through epigenetic changes[14–16].

Obesity is a multifactorial disorder that is believed to occur as a result of gene- environment interaction. Early evidence of the combined influence of genetics and the environment on obesity comes from heritability studies involving twins in different environments [17]. Environmental changes like sedentary lifestyles, high-calorie diets, and weight-promoting drugs have significantly increased obesity rates. However, genetic factors also play a role in an individual's response to an 'obesogenic' environment [18]. Despite known genetic susceptibility, much of obesity's heritability remains unknown [19]. Existing evidence indicates that lifestyle factors can significantly modify the impact of gene variants predisposing individuals to obesity [17]. It may be presumed that the social changes in terms of food abundance and sedentary lifestyle may expand the impact of environment (and consequently minimize the impact of genes) in the establishment of the obese phenotypes. Intriguingly, the proportion of variation in BMI ascribable to genetic variation is elevated among individuals living in the modern ‘obesogenic’ environment[20, 21]. Additionally, the estimates of obesity heritability increase with increased severity of obesity status[22].

Obesity is classified into two main genetic categories: monogenic and polygenic. Monogenic obesity is rare, severe, and early-onset, caused by single-gene defects and follows Mendelian inheritance, with a strong genotype–phenotype correlation. Polygenic obesity involves multiple polymorphisms, each with a small effect [23, 24]. The distinction between these types is blurring as they share biochemical pathways and they are now considered to lie on a spectrum[24].

Despite identifying over 1,000 loci through genome-wide association studies (GWAS), the specific genes, tissues, and mechanisms involved are largely unknown[25]. These variants do not fully account for the heritability of obesity, estimated at 40% to 70%, leaving much of the genetic susceptibility unexplained [19].

The possible role of epigenetics in illustrating the unraveled heritability is being considered, possibly through mediating the gene-environment interactions that underlie the rise of obesity prevalence and its related comorbidities [26]. Epigenetics involves heritable changes in gene expression without alterations in the DNA sequence and includes mechanisms such as DNA methylation, histone modification, integration of histone variants, ATP-dependent chromatin remodeling, and the actions of noncoding RNAs (ncRNAs) [27]. The dynamic nature of the epigenome may better explain the dramatic increase in obesity prevalence over the past 40 to 50 years, a period insufficient for significant new DNA sequence variations leading to obesity[28].

Approximately 1.2% of the human genome encodes proteins, while the remainder is considered non-coding. Most of the mammalian genome is transcribed into RNA, but these RNAs are not translated into proteins and are classified as noncoding RNAs [29]. Noncoding RNAs include housekeeping structural RNAs, such as ribosomal RNA (rRNA), transfer RNA (tRNA), small nuclear RNA (snRNA), small nucleolar RNA (snoRNA), and telomerase RNA (TER). On the other hand, regulatory ncRNAs are responsible for gene expression regulation at epigenetic, transcriptional, and post-transcriptional levels. These regulatory ncRNAs are classified into short regulatory RNAs (including micro-RNAs (miRNAs), small interfering RNAs (siRNAs), and PIWI-interacting RNAs (piRNAs)) and the less well-characterized long non-coding RNAs (lncRNAs) [30].

Studies have identified the involvement of multiple lncRNAs in regulating body weight homeostasis, obesity-related complications, adipogenesis, and the metabolism and function of white and brown adipose tissues[31]. A significant decrease in the circulating levels of lncRNA-p5549, lncRNA-p21015, and lncRNA-p19461 was observed in the blood of obese human subjects compared to those of average weight[32].

Epigenetic changes, including alterations in DNA methylation of specific genes, have been observed following MBS. Restoration of normal methylation levels after MBS was detected in multiple studies[33–36]. Transgenerational research indicated that the methylome of children born to obese females before undergoing MBS differed from that of siblings born after the surgery, suggesting that maternal surgery can alter the epigenetics of offspring. These differentially methylated genes were linked to leptin signaling, insulin receptor signaling, and type 2 diabetes signaling [37].

Regarding other epigenetic mechanisms like histone modification and chromatin remodeling, no studies have yet assessed their influence by MBS.[38]. Only few studies have investigated changes in lncRNA expression following such surgery, mostly using animal models[39, 40]. This study aims to evaluate whether MBS as a weight-loss therapy can reverse the altered levels of specific lncRNAs, namely lncRNA-p5549 and lncRNA-p19461, in patients with morbid obesity. To our knowledge, this approach has not been applied in previous studies.

## Patients and Methods

This prospective study was conducted on thirty-four consecutive patients with obesity, scheduled for MBS and who agreed to participate in this study. Sample size was calculated using G. Power software®, considering a power of 80%, a confidence level of 95%, and mean levels of circulating lncRNA p5549 of 0.15 ± 0.08 versus 0.19 ± 0.1 after intervention. Exclusion criteria included patients who had undergone previous MBS and the presence of associated malignancy. Informed consent was obtained from all participants, and the Institutional Review Board (IRB) approved the study.

### Data Collection

Preoperatively, we collected and analyzed the baseline characteristics of the patients, along with the incidence of obesity-associated medical problems. The following clinical and physical parameters were recorded on the day of surgery and again 12 weeks post-surgery: weight, height, BMI, hip circumference, waist circumference, and waist-to-hip ratio. Postoperative weight loss was calculated as a percentage of total weight loss and percentage of excess BMI lost (%EBL).

Preoperative laboratory investigations were completed. Additionally, two milliliters of venous blood were collected via venipuncture for RNA extraction on the day of surgery and again 12 weeks post-surgery, under aseptic conditions, using EDTA tubes. No patients were lost to follow-up.

### Metabolic/ bariatric surgery (MBS)

Patients underwent either Laparoscopic Sleeve Gastrectomy (LSG) or Roux-en-Y Gastric Bypass (RYGB). Any perioperative complications were documented.

### Assessment of circulating lncRNA- p5549 and p19461

RNA extraction and quantitative RT-PCR measurement of lncRNA-p5549 and lncRNA-p19461 were conducted using Syber-Green Realtime quantitative PCR. Plasma was extracted by centrifuging blood samples at 3000 rpm for five minutes twice. Total RNA was extracted from 200 µl of plasma samples using the miRNeasy Mini Kit from Qiagen, following the manufacturer's instructions. The quality and quantity of the extracted RNA were assessed with a Nanodrop spectrophotometer (Thermo Scientific). RNA samples were aliquoted and stored at −80˚C until used for complementary DNA (cDNA) synthesis. cDNA synthesis was carried out using the RevertAid First Strand cDNA Synthesis Kit from Thermo Scientific™, following the manufacturer's instructions. Each PCR experiment included a negative control (No Template Control) containing the same reaction components, except RNA, to exclude any contamination. The cDNA was stored at −20˚C until required.

Real-time quantitative PCR was performed using Maxima SYBR Green qPCR Master Mix, following the manufacturer’s instructions. The purity of the amplification products was verified at the end of each cycle by analyzing melting curves. The expression levels of lncRNAs were normalized using the housekeeping gene glyceraldehyde 3-phosphate dehydrogenase (GAPDH) as an internal control for relative expression quantification. The specificity of primers was detected using NCBI primer-BLAST online tool. The relative expression quantification was calculated using Qiagen data analysis center platform and the comparative Cq method (2^-ΔΔCq).

## Statistical Analysis

Data were fed to the computer and analyzed using IBM SPSS software package version 20.0. (Armonk, NY: IBM Corp). Continuous data were tested for normality by the Shapiro–Wilk test. Quantitative data were expressed as range (minimum and maximum), mean, standard deviation, and median. To compare preoperative and postoperative normally distributed quantitative variables, the paired t-test was used. For quantitative variables that were not normally distributed, the Wilcoxon signed ranks test was used. The significance of the obtained results was judged at the 5% level.

## Results

This prospective study included thirty-four patients with obesity who were scheduled for metabolic/bariatric surgery. Twenty-six patients (76.5%) underwent Laparoscopic Sleeve Gastrectomy (LSG), while the remaining eight (23.5%) underwent Roux-en-Y Gastric Bypass (RYGB). Table 1 illustrates the baseline characteristics, and the changes observed in anthropometric parameters 12 weeks after metabolic/bariatric surgery. All postoperative anthropometric measurements showed a statistically significant reduction compared to the preoperative measurements. The incidence and changes in obesity-associated medical conditions are presented in Tables 2 and 3, where a statistically significant improvement or remission was observed in most of these conditions.

**Table 1:** Changes in Anthropometric measurements after MBS (*n* = 34)

	LSG Surgery (*n* = 26)		RYGB Surgery (*n* = 8)	
	Preoperative	Postoperative	Preoperative	Postoperative
Weight (kg)				
Mean ± SD	116 ± 17.37	91.69 ± 14.88	131 ± 32.18	105.3 ± 24.16
Median (Min. – Max.)	113.5 (90 – 154)	90 (68 – 120)	133 (95 – 177)	105 (80 – 141)
	*p* < 0.001^*^		*p* < 0.001^*^	
BMI (kg/m^2^)				
Mean ± SD	42.73 ± 5.57	33.76 ± 4.83	46.99 ± 9.94	37.73 ± 7.55
Median (Min. – Max.)	41.5 (35.16 – 56.6)	32.4 (26.6 – 44.1)	44.95 (38.5 – 68.28)	35.95 (31.3 – 54.4)
	*p* < 0.001^*^		(*p* < 0.001^*^	
Waist circumference (Cm)				
Mean ± SD	116.8 ± 12.89	99.11 ± 13.20	120.5 ± 22.43	102.6 ± 22.82
Median (Min. – Max.)	117 (93 – 143)	98.25 (80 – 123)	121.5 (90 – 150)	107 (65 – 132)
	*p* < 0.001^*^		*p* = 0.004^*^	
Hip circumference (Cm)				
Mean ± SD	136.6 ± 14.47	122.02 ± 15.37	138.6 ± 20.36	124.13 ± 16.63
Median (Min. – Max.)	138.3 (116 – 183)	122 (98 – 165)	136 (117 – 180)	122 (104 – 153)
	*p* < 0.001^*^		*p* < 0.001^*^	
W/H ratio				
Mean ± SD	0.86 ± 0.09	0.82 ± 0.09	0.87 ± 0.10	0.82 ± 0.11
Median (Min. – Max.)	0.85 (0.72 – 1.04)	0.80 (0.62 – 1.02)	0.91 (0.66 – 0.98)	0.85 (0.63 – 0.93)
	*p* = 0.033^*^		*p* = 0.176	

SD: Standard deviation

*p*: *p* value for comparing between Preoperative and Postoperative

^*^: Statistically significant at *p* ≤ 0.05

**Table 2:** The incidence and changes in Hypertension and T2DM (*n* = 34)

	No. (%)
Hypertension [preoperative]	6/34 (17.6%)
Postoperative	
Improvement	5/6 (83.33%)
Remission	1/6 (16.66%)
T2DM [preoperative]	8/34 (23.52%)
Postoperative	
Improvement	5/8 (62.5%)
Remission	3/8 (37.5%)

**Table 3:** The incidence and changes in diabetic profile and lipid profile (*n* = 34)

	Total Sample (*n* = 34)		LSG Surgery (*n* = 26)		RYGB Surgery (*n* = 8)	
	Preop	Postop	Preop	Postop	Preop	Postop
Fasting glucose (mg/dl)						
Mean ± SD	99.84 ± 16.84	77.64 ± 12.51	97.32 ± 16.97	77.26 ± 13.74	108 ± 14.46	78.88 ± 7.83
Median (Min. – Max.)	97 (76 – 138)	78 (50 – 115)	96 (76 – 138)	78 (50 – 115)	107 (92 – 137)	78.5 (67 – 93)
	*p* < 0.001^*^		*p* < 0.001^*^		*p* = 0.002^*^	
HbA1c (%)						
Mean ± SD	5.83 ± 1.02	5.34 ± 0.31	5.62 ± 0.49	5.33 ± 0.33	6.51 ± 1.83	5.39 ± 0.25
Median (Min. – Max.)	5.6 (4.9 – 10.9)	5.3 (4.6 – 6)	5.6 (4.9 – 6.9)	5.3 (4.6 – 6)	6.15 (5.3 – 10.9)	5.3 (5.1 – 5.7)
	*p* = 0.007^*^		*p* = 0.001^*^		*p* = 0.126	
Cholesterol (mg/dl)						
Mean ± SD	202.2 ± 43.34	182.1 ± 35.22	205.2 ± 42.58	185.4 ± 34.6	192.1 ± 47.19	171.5 ± 37.47
Median (Min. – Max.)	200.5(139 – 336)	176.5(121 – 279)	201(141 – 336)	183(121.7 – 279)	176.5(139 – 294)	165.5(121 – 246)
	*p* = 0.001^*^		*p* = 0.002^*^		*p* = 0.167	
Triglycerides (mg/dl)						
Mean ± SD	129.4 ± 56.89	105.4 ± 31.99	127.4 ± 59.17	104.8 ± 32.01	135.8 ± 51.89	107.25 ± 34.04
Median (Min. – Max.)	119.3 (58 – 274)	99 (58 – 190)	105.8 (58 – 274)	102.5 (58 – 190)	151.5 (71 – 187)	92.5 (64 – 170)
	*p* = 0.007^*^		*p* = 0.027^*^		*p* = 0.183	
LDL (mg/dl)						
Mean ± SD	129.2 ± 38.12	116.2 ± 32.49	128.7 ± 37.47	117.5 ± 31.57	130.6 ± 42.82	112 ± 37.29
Median (Min. – Max.)	118.5 (67 – 226)	113 (47 – 187)	118.5 (67 – 226)	118.5 (47 – 182)	120.5 (80 – 220)	106 (57 – 187)
	*p* = 0.001^*^		*p* = 0.005^*^		*p* = 0.093	
HDL (mg/dl)						
Mean ± SD	49.70 ± 10.38	48.35 ± 10.78	51.8 ± 10.71	49.69 ± 9.6	42.88 ± 5.38	44 ± 13.79
Median (Min. – Max.)	45.25 (33 – 73)	48.50 (28 – 73)	50.5 (36 – 73)	50.5 (33 – 72)	43.5 (33 – 52)	43.5 (28 – 73)
	*p* = 0.416		*p* = 0.225		*p* = 0.833	

*SD* Standard deviation,*t* Paired t-test, *Z* Wilcoxon signed ranks test

*p*: *p* value for comparing between Preoperative and Postoperative

^*^: Statistically significant at *p* ≤ 0.05

The first column demonstrates the effect of MBS collectively in the total number of patient (n = 34), while in the other two columns patients are divided according to the surgery type whether LSG (*n* = 26), or RYGB (*n* = 8)

**Fig. 1:**
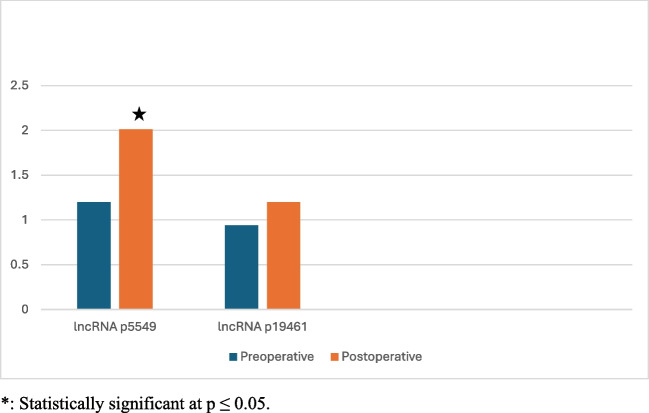
Serum lncRNA p19461 and lncRNA p5549 before and after bariatric surgery

The expression levels of long non-coding RNA p5549 (lncRNA-p5549) in serum significantly increased in postoperative samples compared to preoperative samples, with a fold increase of 4.63 ± 7.68 (p = 0.014), as shown in Table 4 and Fig. 1. However, the postoperative increase in the expression levels of long non-coding RNA p19461 (lncRNA-p19461) in serum was not statistically significant.

**Table 4:** Comparison between preoperative and postoperative serum levels of ncRNA- p19461 and lncRNA-p5549

lncRNA- p5549	Before surgery	After surgery	p
**Total sample (n = 34)**			
Median (Min. – Max.)	1.20 (0.05 – 7.97)	2.01 (0.24 – 24.35)	0.014*
**Fold**	**4.63 ± 7.68**		
**LSG Surgery (n = 26)**			
Mean ± SD	1.50 ± 1.54	2.57 ± 2.21	0.040^*^
Median (Min. – Max.)	1.20 (0.05 - 7.97)	1.84 (0.24 - 8.54)	
**Fold**	**4.56 ± 8.20**		
**RYGB Surgery (n = 8)**			
Mean ± SD	1.36 ± 0.85	5.36 ± 8.14	0.208
Median (Min. – Max.)	1.23 (0.15 - 2.35)	2.56 (0.30 - 24.35)	
**Fold**	**4.86 ± 6.14**		
**lncRNA- p19461**	**Before surgery**	**After surgery**	**p**
**Total sample (n = 34)**			
Median (Min. – Max.)	0.94 (0.12 – 12.22)	1.20 (0.04 - 22.13)	0.561
**Fold**	**4.43 ± 10.99**		
**LSG Surgery (n = 26)**			
Median (Min. – Max.)	0.94 (0.24 - 12.22)	1.16 (0.04 - 22.13)	0.493
**Fold**	**5.35 ± 12.46**		
**RYGB Surgery (n = 8)**			
Median (Min. – Max.)	0.75 (0.12 - 6.05)	1.25 (0.15 - 2.42)	0.674
**Fold**	**1.43 ± 1.31**		

^*^Statistically significant at *p* ≤ 0.05

A negative correlation was observed between serum levels of lncRNA-p5549 and both waist circumference and waist-to-hip ratio, as displayed in Table 5.

**Table 5:** Correlation of serum lncRNA- p5549 with waist circumference, and waist to hip ratio

	Postoperative lncRNA- p5549	
	rs	p
Waist circumference	−0.464*	0.006*
W/H ratio	−0.346*	0.045*

## Discussion

Most patients in this study were female (88.23%), consistent with a previous study on gender disparity among patients undergoing metabolic/bariatric surgery, which reported that 80% of such patients were female[41]. The factors underlying this disparity include gender-based differences in the perception of body weight, more pronounced psychosocial obesity-related distress among women, and culturally accepted male body types that permit men to have a high BMI without social stigmatization[42].

In patients with obesity, losing 5 to 10% of body weight is sufficient to prompt clinically relevant improvements in obesity-related risk factors. However, it is critical to sustain the weight loss to preserve the health benefits[43]. In the present study, postoperative weight loss was satisfactory, with the percentage of total weight loss (TWL) and the percentage excess BMI lost (%EBL) measuring at 20.64% ± 3.34% and 51.71% ± 14.03%, respectively. Our results align with those of Grover et al., who considered a total weight loss (TWL) of ≥ 20% as indicative of successful outcomes in a trial aimed at standardizing results [44]. Additionally, Philouze et al. found that a %EBL exceeding 20.1% at three months post-surgery indicates successful surgery and serves as a predictive factor for long-term results after sleeve gastrectomy. They noted that the rate of %EBL is highest during the initial three months post-surgery, during which patients typically achieve most of their %EBL[45]. Furthermore, the significant reductions in hip circumference, waist circumference, and hip-to-waist ratio after surgery (p < 0.001, p < 0.001, and p < 0.01, respectively) are consistent with the findings of a study conducted by Wei et al., which also demonstrated statistically significant reductions in these measurements three months after weight reduction surgery[46].

Among the six patients with associated hypertension, 5 (83.33%) experienced improvement, while 1 (16.67%) achieved remission three months following surgery. Pedersen et al. reported a significant reduction in systolic and mean arterial pressure in both hypertensive and normotensive patients as early as ten days after surgery, before any substantial weight loss[47].

Another study conducted by Ahmed et al., which examined 100 hypertensive and normotensive patients with obesity, observed a reduction in both systolic and diastolic blood pressure one week after RYGB[48]. A retrospective study analyzing blood pressure changes one month after RYGB in 95 hypertensive patients found a reduction in systolic blood pressure from 140 ± 17 mmHg before surgery to 123 ± 18 mmHg, and in diastolic blood pressure from 80 ± 11 mmHg to 75 ± 10 mmHg[49]. The mechanism through which MBS reduces blood pressure before significant weight loss occurs remains unclear and may also be related to epigenomic regulation, which warrants further investigation in an extended study. It has been suggested that the blood pressure-lowering effect of RYGB is not solely attributed to weight reduction but also to the influence exerted by altered intestinal hormones or peptides on renal function through an 'entero-renal' axis[47].

T2DM improved in 5 (62.5%) and was remitted in 3 (37.5%) of the 8 patients with preoperative diabetes mellitus. MBS has demonstrated a drastic effect on glycemic control preceding weight loss[50], possibly through the reduction of insulin resistance and enhancement of β-cell function immediately after MBS[51].

Regarding the lipid profile, the significant reductions in total cholesterol (TC), triglycerides (TG), and LDL levels mirror the findings of Milone et al. [52]. Another study demonstrated significant decreases in TC, TG, and LDL three months after RYGB, with HDL showing a significant increase at 12 months post-surgery[53]. Piché et al. also observed significant improvements in the lipid profile before substantial weight loss occurred[54].

To the best of our knowledge, this study's approach of comparing differences in lncRNA-p19461 and lncRNA-p5549 expression before and after MBS is among the first to explore the epigenomic regulation of weight loss following bariatric surgery.

lncRNAs are involved in all indispensable processes in the cell, including DNA replication, transcription, mRNA splicing, protein translation, and post-translational modification, in addition to the regulation of gene expression at all levels, from chromatin remodeling to adjusting the stability of mRNAs encoding proteins within the cytoplasm[31]. Some lncRNAs function locally by controlling the expression of nearby genes in cis, whereas others exert their effects in trans at several different loci throughout the genome[30].

The actions of lncRNAs extend beyond chromatin. Some lncRNAs exert a sponge-like effect on regulatory elements such as miRNAs, which lessens their effect on target mRNAs. Others alter the cell's ability to identify mRNA molecules, which in turn affects their translation efficiency, stability, and cellular confinement[30]. Despite the several examples illustrating the biological roles of lncRNAs, the mechanisms of action of the greater bulk of them remain undisclosed[30].

Sun et al. assessed the genome-wide circulating lncRNA expression levels and discovered that lncRNA-p19461 and lncRNA-p5549 were significantly lower in individuals with obesity compared to individuals with normal weight[32]. lncRNA-p5549 showed a significant decrease in a selected cohort of six patients with breast cancer and obesity compared to six patients with breast cancer and normal weight[55].

The study by Sun et al. reported a significant increase in the expression level of lncRNA-p19461 in eight individuals with obesity following weight loss induced by a 12-week very low carbohydrate diet. Meanwhile, the increase in lncRNA-p5549 expression was not statistically significant[32].

In our study, lncRNA-p5549 was found to be significantly increased after MBS, whereas the increase in lncRNA-p19461 expression was not statistically significant. We speculate that the epigenetic changes resulting from diet-induced weight loss might differ from those resulting from bariatric surgery-induced weight loss.

Very limited data are available on the expression and biological functions of lncRNA-p5549; however, it was found to be associated with thymine DNA glycosylase enzyme (TDG), which binds to the enzymes of DNA methylation to maintain the methylation signature in a normal status, a feature that might represent an epigenetic mechanism of controlling metabolic homeostasis[32]. TDG is also involved in transcriptional regulation and DNA repair. lncRNA-p5549 was also found to be involved in multiple pathways, including pathways of fatty acid metabolism, toll-like receptors (TLR), mitogen-activated protein kinase (MAPK), antigen processing and presentation, and the immune network for IgA production in the intestine. It was also found to interact with several mRNAs in meaningful way[32]. Meanwhile, lncRNA-p19461 may protect mitochondrial function through regulation of CHCHD3, accelerating fat oxidation and reducing the incidence rate of obesity. The downregulation of lncRNA-p19461 in individuals with obesity may lead to reduced energy consumption[32]. How the restoration of lncRNA-p5549 expression levels is related to the metabolic changes after MBS remains to be elucidated and needs to be further investigated.

## Limitations

The study was conducted over a relatively short period. We plan to assess the long-term effect of MBS on these lncRNAs in a future extended study. A larger sample size might be needed to confirm and validate the results. Further studies are needed to assess the mechanism of involvement of lncRNA-p5549 in weight loss and enhancement in obesity-associated medical problems.

## Conclusion

Bariatric surgery-induced weight reduction can reverse the dysregulation of lncRNA-p5549 expression in individuals with obesity. While lncRNA-p19461 expression levels increase after MBS, the increase is not statistically significant. The epigenetic changes resulting from diet-induced weight loss might differ from those resulting from bariatric surgery-induced weight loss however, a two- armed study is needed to validate such speculation.

## Data Availability

The data that support the findings of this study are not openly available due to reasons of sensitivity and are available from the corresponding author upon reasonable request.

## Abbreviations

lncRNA: Long noncoding ribonucleic acid
BMI: Body mass index
ORCs: Obesity related complications.
T2D: Type 2 diabetes.
GWAS: Genome- wide association studies.
DNA: Deoxy ribonucleic acid.
ATP: Adenosine triphosphate.
ncRNAs: Noncoding ribonucleic acids.
RNA: Ribonucleic acids.
mRNA: Messenger ribonucleic acids.
OMM: Obesity- management medications
LSG: Laparoscopic sleeve gastrectomy.
RYGB: Roux-en-Y gastric bypass.
TDG: Thymine DNA glycosylase
MAPK: Mitogen-activated protein kinase
cDNA: Complementary deoxy ribonucleic acid.
PCR: Polymerase chain reaction.
qPCR: Quantitative polymerase chain reaction.
GAPDH: Glyceraldehyde 3 phosphate dehydrogenase.
IQR: Interquartile range.
W/H ratio: Waist / hip ratio.
%EBL: Percentage of excess body mass index lost.
HbA1c: Glycated hemoglobin.
LDL: Low- density lipoprotein.
TLR: Toll- like receptors
IgA: Immunoglobulin A
GLP-1: Glucagon- like peptide- 1
GIP: Glucose-dependent insulinotropic polypeptide
